# *NOS2 *polymorphisms associated with the susceptibility to pulmonary arterial hypertension with systemic sclerosis: contribution to the transcriptional activity

**DOI:** 10.1186/ar1984

**Published:** 2006-07-03

**Authors:** Yasushi Kawaguchi, Akiko Tochimoto, Masako Hara, Manabu Kawamoto, Tomoko Sugiura, Yasuhiro Katsumata, Jun Okada, Hirobumi Kondo, Mitsuo Okubo, Naoyuki Kamatani

**Affiliations:** 1 Institute of Rheumatology, Tokyo Women's Medical University, Tokyo, Japan; 2 Department of Internal Medicine, Kitasato University School of Medicine, Sagamihara, Japan; 3 Transfusion Medicine and Cell Therapy, Saitama Medical School, Kawagoe, Japan

## Abstract

Systemic sclerosis (SSc) is a connective tissue disease characterized by tissue fibrosis. One of several complications of SSc, pulmonary arterial hypertension (PAH) can be refractory to treatment, both novel and established. In the present study we investigated the ratio of circulating nitric oxide to endothelin-1 in patients with both SSc and PAH, and determined whether polymorphisms in *NOS2 *(the nitric oxide synthase 2 gene) are associated with susceptibility to PAH. Endothelin-1 in plasma and nitric oxide metabolites (nitrate and nitrite) in serum were measured. The nitric oxide/endothelin-1 ratio was significantly lower in patients with both SSc and PAH than in patients with SSc only or in healthy control individuals. We confirmed the presence of two single nucleotide polymorphisms at positions -1,026 and -277 and a pentanucleotide repeat (CCTTT) at -2.5 kilobases. There were significant differences in single nucleotide polymorphisms between patients with SSc who had PAH and those who did not, and between patients with both SSc and PAH and healthy control individuals. The CCTTT repeat was significantly shorter in patients with both SSc and PAH than in patients with SSc only or in healthy control individuals. Transcriptional activity were analyzed using the luciferase reporter assay. The transcriptional activity of *NOS2 *was much greater in fibroblasts transfected by a vector with a long allele of the CCTTT repeat than in those transfected by a vector with a short allele. Polymorphisms in the *NOS2 *gene are associated with transcriptional activity of the *NOS2 *gene and with susceptibility to SSc-related PAH.

## Introduction

Systemic sclerosis (SSc) is an autoimmune disease of unknown aetiology that is characterized by extensive fibrosis of skin and visceral organs, and dysfunction of vascular tone [1]. In its more severe forms, cardiac involvement and respiratory involvement are the most significant determinants of outcome [2]. In particular, pulmonary hypertension is a fatal complication in both diffuse and limited cutaneous SSc [3]. Pulmonary hypertension is generally divided into four major categories: pulmonary arterial hypertension (PAH), pulmonary hypertension associated with left-sided heart disease, pulmonary hypertension associated with lung disease or hypoxaemia, and pulmonary hypertension due to chronic thrombotic or embolic disease [4]. A major part of pulmonary hypertension as it pertains to SSc corresponds with the pathophysiology of PAH, a disease of the small pulmonary arteries characterized by vascular proliferation, vasoconstriction, remodelling of the pulmonary vessel wall and thrombosis in vessels.

Vasodilators such as nitric oxide (NO) and prostacyclin, along with prolonged overexpression of vasoconstrictors such as endothelin (ET)-1, not only affect vascular tone but also promote vascular remodelling, both of which have been implicated in the pathogenesis of PAH [5-12]. Previous studies identified high levels of ET-1 in the plasma of patients with SSc, especially in those with SSc complicated by PAH [13,14]. However, reported levels of circulating NO in patients with SSc are inconsistent, with several studies [15-18] finding increased levels of NO in patients with SSc and others [19,20] finding low levels, similar to those in healthy individuals. In our previous study [21] NO levels were markedly elevated in patients with early-stage diffuse cutaneous SSc, especially when the SSc was accompanied by active alveolitis, but concentrations of NO in serum were low in late-stage limited cutaneous SSc. No patients suffered the complication of PAH in that study. Characteristic levels of NO and NO/ET-1 ratio in patients with both SSc and PAH remain to be established.

NO is an endothelial-derived relaxing factor that is synthesized from L-arginine by nitric oxide synthase (NOS) [22]. Three isoforms of NOS have been identified [23]: NOS-1 (neuronal NOS), NOS-2 (inducible NOS) and NOS-3 (endothelial NOS). NOS-2 is the major source of NO production in conditions involving exposure to cytokines; this is because it is induced by a variety of cell types, including the proinflammatory cytokines interleukin-1, tumour necrosis factor-α, interferon-γ, and ET-1 [24].

Two randomized, double-blind, placebo-controlled trials [25,26] evaluated the efficacy of the ET receptor antagonist bosentan in patients with PAH that was either primary or associated with SSc. Another therapeutic strategy in PAH is to increase the activity of endogenous NO, which enhances NO-dependent cGMP-mediated pulmonary vasodilatation through inhibition of the breakdown of cGMP by phosphodiesterase type 5 [27]. Although long-term inhaled NO therapy has shown only a small benefit in patients with PAH [28], phosphodiesterase type 5 inhibitors (for example, sildenafil) have been found to improve pulmonary artery pressure in patients with PAH [29].

Because these novel therapies were developed to prolong survival and improve patients' quality of life, we speculate that an imbalance between ET-1 and NO is key to the pathogenesis of SSc complicated by PAH. Polymorphisms in the *NOS2 *gene promoter are thought to regulate its transcription activity, which is reportedly associated with susceptibility to type 1 diabetes [30] and atopy [31] and with protection against malaria [32]. In the present study we determined the levels of ET-1 and NO in blood from patients with SSc with or without PAH, and we investigated the association between gene polymorphisms in *NOS2 *and susceptibility to PAH.

## Materials and methods

### Study patients

Twenty patients with SSc complicated by PAH were recruited. All had been admitted to Aoyama Hospital of Tokyo Women's Medical University or Kitasato University Hospital. As a disease control group, 58 patients with SSc but not PAH were selected from patients admitted to Aoyama Hospital. Detailed clinical characteristics of all patients are shown in Table 1. All patients with SSc were of Japanese origin, met the criteria established by the American College of Rheumatology for SSc [33], and were classified as having either diffuse or limited cutaneous SSc according to the classification proposed by LeRoy and coworkers [34]. Ninety-five DNA samples were obtained from healthy volunteers who were unrelated individuals of Japanese origin. All DNA samples were collected, with approvals granted by the appropriate ethical committees of Tokyo Women's Medical University, Kitasato University School of Medicine, and Saitama Medical School.

We identified the presence of a complication of PAH in the following manner. All patients with SSc were first evaluated by Doppler echocardiography, and then cardiac catheterization was performed when right ventricular systolic pressure was greater than 30 mmHg, based on Doppler echocardiography. PAH was diagnosed in patients with SSc who satisfied the modified US National Institutes of Health criteria for PAH after cardiac catheterization [35], specifically mean pulmonary artery pressure above 25 mmHg at rest or 30 mmHg after exercise, with normal pulmonary artery wedge pressure. The complication of pulmonary fibrosis was identified using high-resolution computed tomography of the chest. Patients with the following complications were excluded: severe pulmonary fibrosis, with functional vital capacity below 70%; left-sided heart disease; chronic thrombotic or embolic disease; renal failure, including a history of scleroderma renal crisis; hypertension; and diabetes.

### Measurement of plasma endothelin-1 and serum nitric oxide levels

Blood samples were obtained from 16 patients with both SSc and PAH and from 26 patients with SSc without PAH who were randomly selected from among patients with SSc who donated DNA samples at the time of admission to Aoyama Hospital with informed consent. No specific diet was given to patients while they were hospitalized. Twenty healthy volunteers (normal control individuals) who had no history of dieting or smoking gave informed consent to participate in the study and gave blood samples. ET-1 levels were measured in plasma using an enzyme-linked immunosorbent assay kit (R&D Systems, Cambridge, MA, USA). Because serum NO is quickly degraded into nitrite and nitrate, we measured the total levels of these NO metabolites as indicators of NO level, using a calorimetric assay kit (Cayman Chemical, Ann Arbor, MI, USA).

### Sequencing the *NOS2 *promoter region

Genomic DNA was extracted from the blood sample using a DNA extraction kit (Qiagen, Valencia, CA, USA). For direct sequencing, PCR was performed to amplify the promoter region of the *NOS2 *gene from -100 to -1,335 bp. The forward and reverse primers were 5'-TCATCCACACATTCACTCAAC-3' and 5'-CCAAAGGGAGTGTCCCCAGCTT-3', respectively. The sequences of the PCR products were analyzed using the ABI Prism 3100 Sequence Detection System (Applied Biosystems, Foster City, CA, USA).

### Haplotype typing in pairs of *NOS2 *polymorphisms

We entered the genotype data into the PENHAPLO computer program, developed by Ito and coworkers [36], to estimate haplotype frequency in the population and to calculate the posterior probability of diplotype distribution for each study subject. This program was designed for haplotype typing using a maximum likelihood estimation method based on the expectation maximization algorithm under the assumption of Hardy-Weinberg equilibrium for the population.

### Analysis of variable numbers of the CCTTT repeat polymorphism of the *NOS2 *promoter region

Genomic DNA was amplified by PCR with the use of a FAM™-labelled sense primer (5'-ACCCCTGGAAGCCTACAACTGCAT-3') and an antisense primer (5'-GCCACTGCACCCTAGCCTGTCTCA-3'). The various alleles were resolved by capillary electrophoresis on an ABI Prism 3100 Genetic Analyzer System (Applied Biosystems). Allele sizes were calculated using the GeneScan Analysis computer program, with a GeneScan™-500 ROX™ size standard (Applied Biosystems) as the internal size standard.

### Analysis of transcriptional activity of *NOS2 *in human fibroblasts

**Figure 1:**
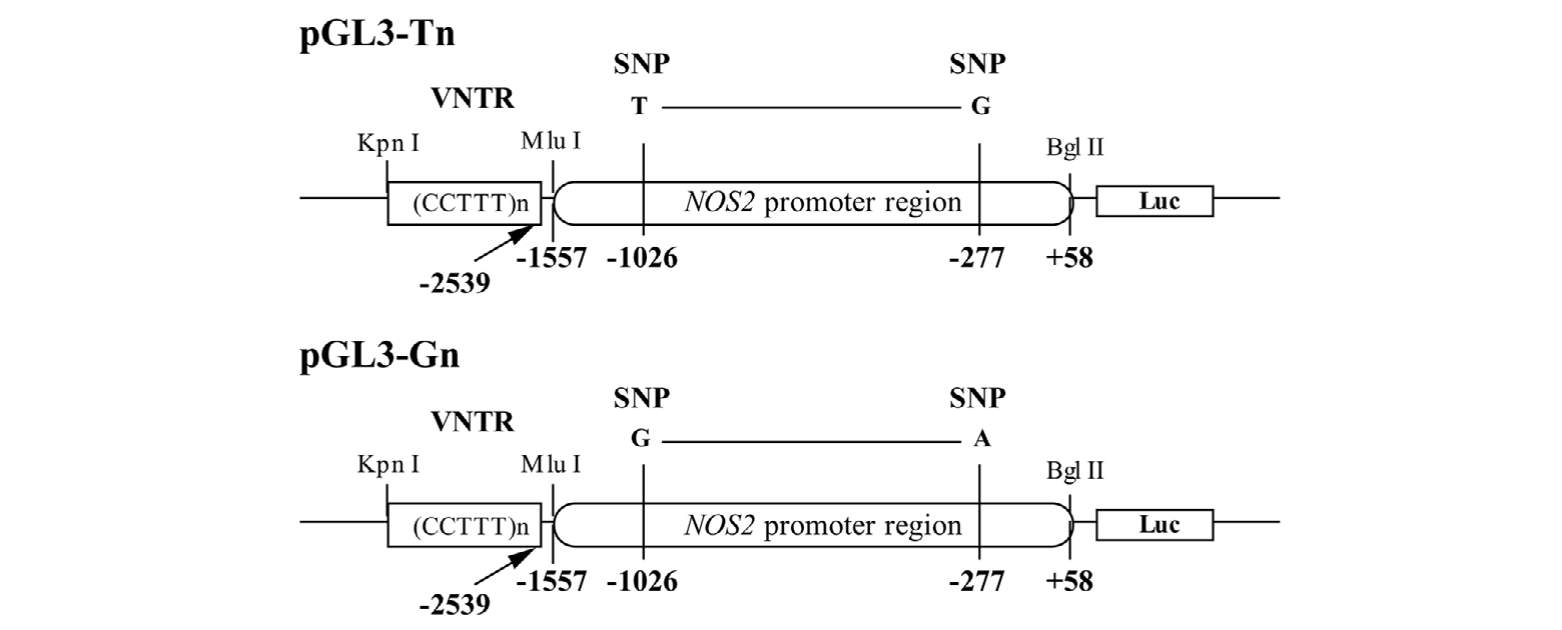
A schematic construct of the promoterless pGL3-basic vector. The luciferase vector (pGL3) was inserted with variable numbers of CCTTT repeats and the 1.5 kb minimal human *NOS2 *promoter region (-1,557 to +58 bp). The promoter region contained either T or G at -1026 bp (referred to as pGL3-T and pGL3-G, respectively). Each vector of pGL3-T and pGL3-G had allele G and allele A at -277 bp, respectively. The 6, 8, 10, 12, and 14 repeats of the pentanucleotide (CCTTT) region were obtained by PCR. The resulting constructs were named pGL3-T6, pGL3-G6, pGL3-T8, pGL3-G8, pGL3-T10, pGL3-G10, pGL3-T12, pGL3-G12, pGL3-T14 and pGL3-G14 and contain 6, 8, 10, 12 and 14 repeats, respectively. bp, base pairs; kb, kilobases; SNP, single nucleotide polymorphism; VNTR, variable numbers of tandem repeat.

The 5' flanking region of the *NOS2 *gene (-1,557 to +58) was prepared by PCR using a set of primers. The forward primer (5'-GATTCTGACTCTTTCCCTGAG-3') is located -1,557 bp from the transcription start site, and the reverse primer (5'-GGAATGAGGCTGAGTTCTCTGCGGC-3') is located +58 bp from the transcription start site. Genomic DNA containing the T/G allele at -1026 bp from the transcription start site of the *NOS2 *gene was used as a PCR template. The PCR product was inserted into a pGL3-Basic Vector (Promega, Madison, WI, USA) that contained the firefly luciferase reporter element, and all constructs were sequenced using the pGL3 forward and reverse sequencing primers. The pGL3 vectors with T or G at -1,026 bp are referred to as pGL3-T and pGL3-G, respectively, as shown in Figure 1. The sequences of inserts of pGL3-T and pGL3-G were confirmed by direct sequencing. Each vector of pGL3-T and pGL3-G had allele G and allele A at -277 bp, respectively. The 6, 8, 10, 12 and 14 repeats of the pentanucleotide (CCTTT) region were obtained by PCR using forward (5'-ACCCCTGGAAGCCTACAACTGCAT-3') and reverse (5'-GCCACTGCACCCTAGCCTGTCTCA-3') primers. The PCR products were cloned into the upstream of the inserted *NOS2 *gene promoter in pGL3-T and pGL3-G. The resulting constructs were named pGL3-T6, pGL3-G6, pGL3-T8, pGL3-G8, pGL3-T10, pGL3-G10, pGL3-T12, pGL3-G12, pGL3-T14 and pGL3-G14, and contain 6, 8, 10, 12 and 14 repeats, respectively.

Human fibroblasts from three healthy individuals were cultured in Dulbecco's modified Eagle's medium (DMEM) with 10% foetal bovine serum (FBS; Sigma, St. Louis, MO, USA). For transient transfections, fibroblasts were cultured in six-well plates with 3 ml Opti-MEM (Invitrogen) containing 4 μg DNA (pGL3 and phRL-TK vectors) and 12 μl Lipofectamine 2000 (Invitrogen). After 4 hours, 3 ml DMEM with 20% FBS in the presence or absence of recombinant interleukin-1β (10 ng/ml; R&D Systems) was added. The medium was changed after 16 hours to DMEM with 10% FBS in the presence or absence of interleukin-1β (5 ng/mL). After an additional 24 hours of culture, the cells were washed twice using cold phosphate-buffered saline and were harvested. Firefly and *Renilla *luciferase activities were measured using the Dual-Glo Luciferase Assay System (Promega). Fibroblasts were cotransfected with a constitutively active *Renilla *luciferase vector (phRL-TK), and firefly luciferase activity was normalized by *Renilla *luciferase activity.

### Statistics

Circulating ET-1 and NO concentrations are given as mean ± standard deviation, and data were compared using the Student's *t *test. We assessed the significance of the -277A/G and -1026G/T single nucleotide polymorphisms (SNPs) by the Fisher exact test. The relationship between the NO/ET-1 ratio and summed CCTTT repeat length was analyzed using linear regression analysis. An allelic distribution of the number of CCTTT repeats was compared using the Mann-Whitney *U *test. *P *< 0.05 was considered statistically significant.

## Results

### Circulating endothelin-1 and nitric oxide concentrations

**Figure 2:**
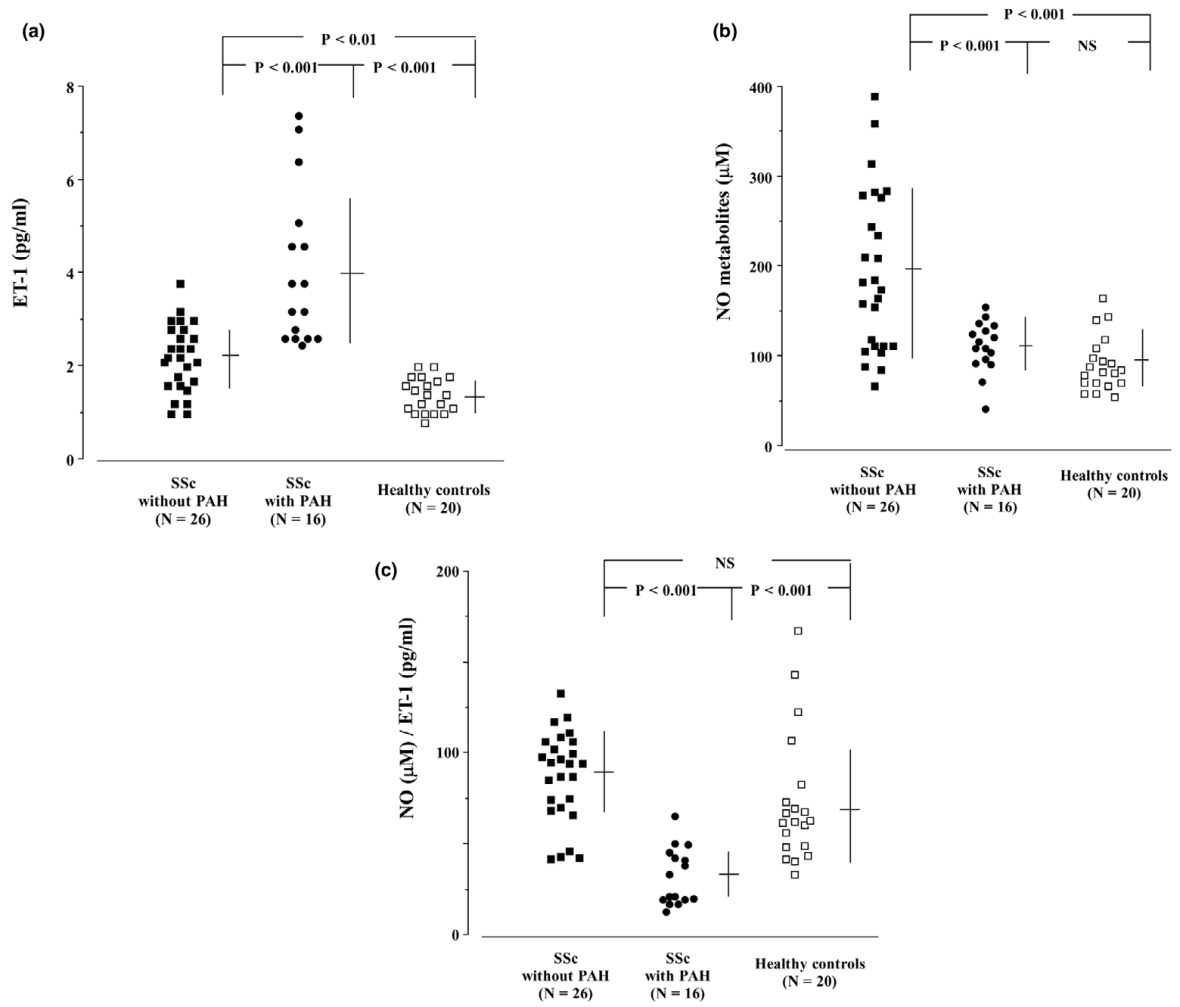
Circulating levels of ET-1 and NO. **(a) **Plasma levels of ET-1 were measured in patients with SSc with or without PAH. **(b) **Serum levels of NO metabolites were measured. **(c) **The ratios of NO/ET-1 are shown. Data are expressed as mean ± standard deviation. ET, endothelin; NO, nitric oxide; NS, not significant; PAH, pulmonary arterial hypertension; SSc, systemic sclerosis.

Plasma ET-1 levels were significantly higher in each SSc group than in healthy control individuals (1.4 ± 0.4 pg/ml), as shown in Figure 2a. Moreover, ET-1 levels in patients with both SSc and PH were significantly higher than in patients with SSc but not PAH (4.1 ± 1.7 versus 2.2 ± 0.8 pg/ml; *P *< 0.001). In contrast, NO levels in patients with both SSc and PAH (114 ± 28 μmol/l) were similar to those in healthy control individuals (95 ± 30 μmol/l), but NO levels in patients with SSc but not PAH (194 ± 89 μmol/l) were significantly higher than in the other two groups (Figure 2b). The NO/ET-1 ratio was significantly lower in patients with both SSc and PAH (32.6 ± 15.7; *n *= 16) than in patients with SSc but not PAH (87.8 ± 25.0; *n *= 26) and healthy control individuals (73.6 ± 35.7; *n *= 20), as shown in Figure 2c.

### Determination of single nucleotide polymorphisms in the *NOS2 *promoter region

**Figure 3:**
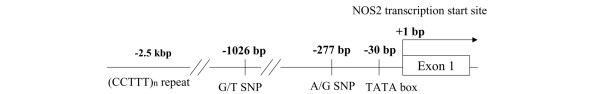
Polymorphisms of the 5' flanking region of the human *NOS2 *gene. TATA sequence begins at position -30 bp from the transcription start site of exon 1. bp, base pairs; SNP, single nucleotide polymorphism.

We genotyped the 78 patients with SSc and the 95 control individuals for the promoter region (-100 to -1335 bp) of the *NOS2 *gene by direct DNA sequencing. We confirmed the presence of two previously reported SNPs at positions -277 and -1026 (Figure 3). The distribution of genotypes is shown in Table 1. The distribution of genotypes at -1026 and -277 was significantly different between patients with SSc who had PAH and those who did not have PAH (both *P *= 0.04, by Fisher's exact test), but there was no difference between patients with SSc who did not have PAH and healthy control individuals in the distribution of genotypes at two SNPs. Between healthy control individuals and patients with both SSc and PAH, there was a significant difference in the distribution of genetypes at -1026 (*P *= 0.02); in contrast, there was no difference at -277 (*P *= 0.053).

### Haplotype typing of the *NOS2 *promoter region

We typed the haplotype of the gene, which consists of two SNPs at positions -1,026 and -277. The two SNPs were found to be in linkage disequilibrium. We identified three haplotypes using genes from patients with SSc and healthy control individuals: GA, GG, and TG (Table 2). The frequency of haplotype GA was significantly higher in patients with both SSc and PAH than in patients with SSc but not PAH and in healthy individuals (*P *= 0.001 and *P *= 0.02, respectively), as shown in Table 2.

### Distribution of variable numbers of tandem repeat in the *NOS2 *promoter region

**Figure 4:**
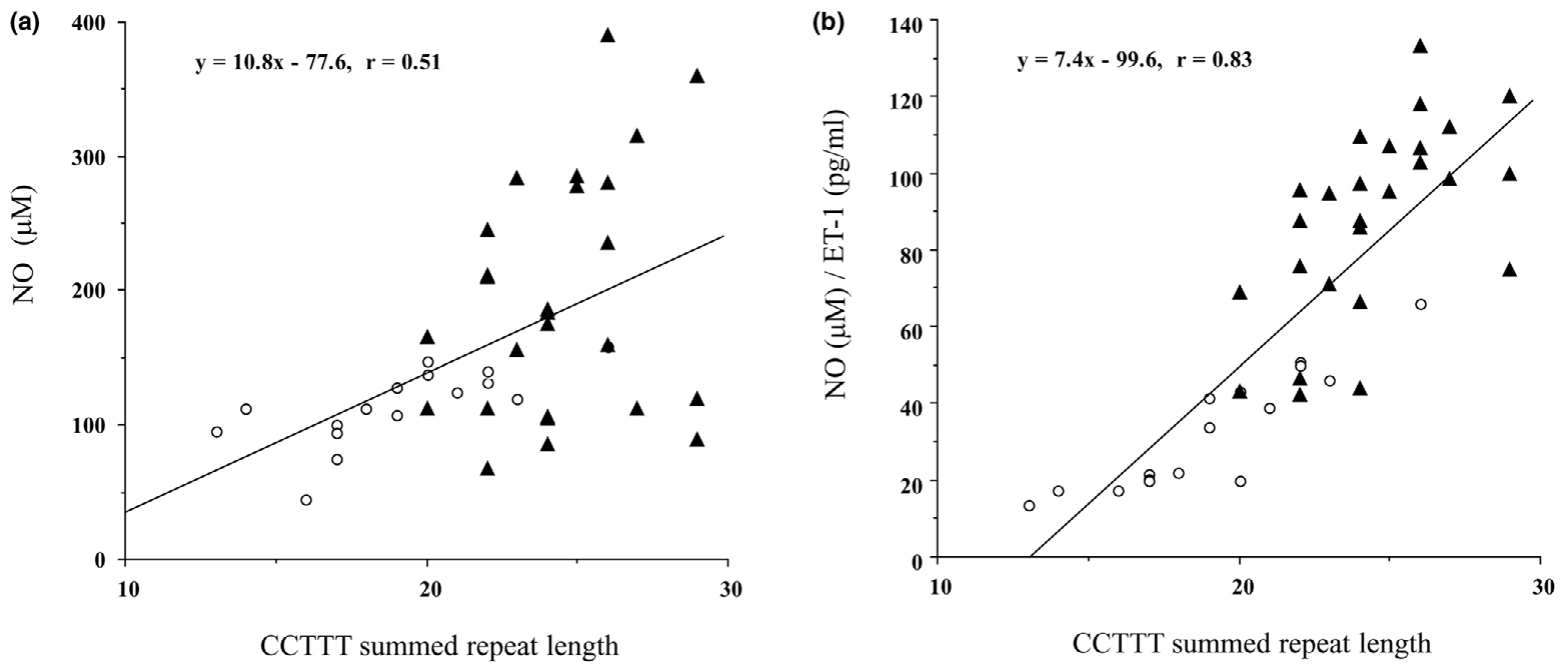
Relationship between NO levels or NO/ET-1 ratios and summed CCTTT repeat length. The association study between summed lengths of the CCTTT repeat and **(a) **serum NO levels and **(b) **NO/ET-1 ratios was performed in 16 patients with SSc with PAH (open circles) and 26 patients with SSc without PAH (black triangles). The results of linear regression analysis of the data are represented by the solid line. Serum NO levels and NO/ET-1 ratios were significantly correlated with summed CCTTT repeat lengths (*r *= 0.51, *P *< 0.01; and *r *= 0.83, *P *< 0.0001, respectively). ET, endothelin; NO, nitric oxide; PAH, pulmonary arterial hypertension; SSc, systemic sclerosis.

The 15 alleles found in the present study had 6–21 repeats, and the distribution was significantly different between patients with SSc and PAH and healthy control individuals (*P *< 0.0001) and between patients with SSc with PAH and those with without PAH (*P *< 0.0001), as shown in Table 3. In contrast, there was no significant difference in distribution between patients with SSc but not PAH and healthy control individuals. If CCTTT repeat length strongly influences *NOS2 *transcription, then we would expect there to be a significant correlation between CCTTT repeat length and serum NO levels or NO/ET-1 ratios. We calculated the number of summed CCTTT repeats and then analyzed the correlation between that number and serum NO levels or NO/ET-1 ratios. As shown in Figure 4, there was a significant correlation between summed repeat length and both serum NO levels (*r *= 0.51, *P *< 0.01; linear regression analysis) and NO/ET-1 ratios (*r *= 0.83, *P *< 0.0001) in all patients with SSc. However, in healthy control individuals we identified no significant correlation (data not shown).

### Effects of *NOS2 *polymorphisms on transcriptional activity of the gene

**Figure 5:**
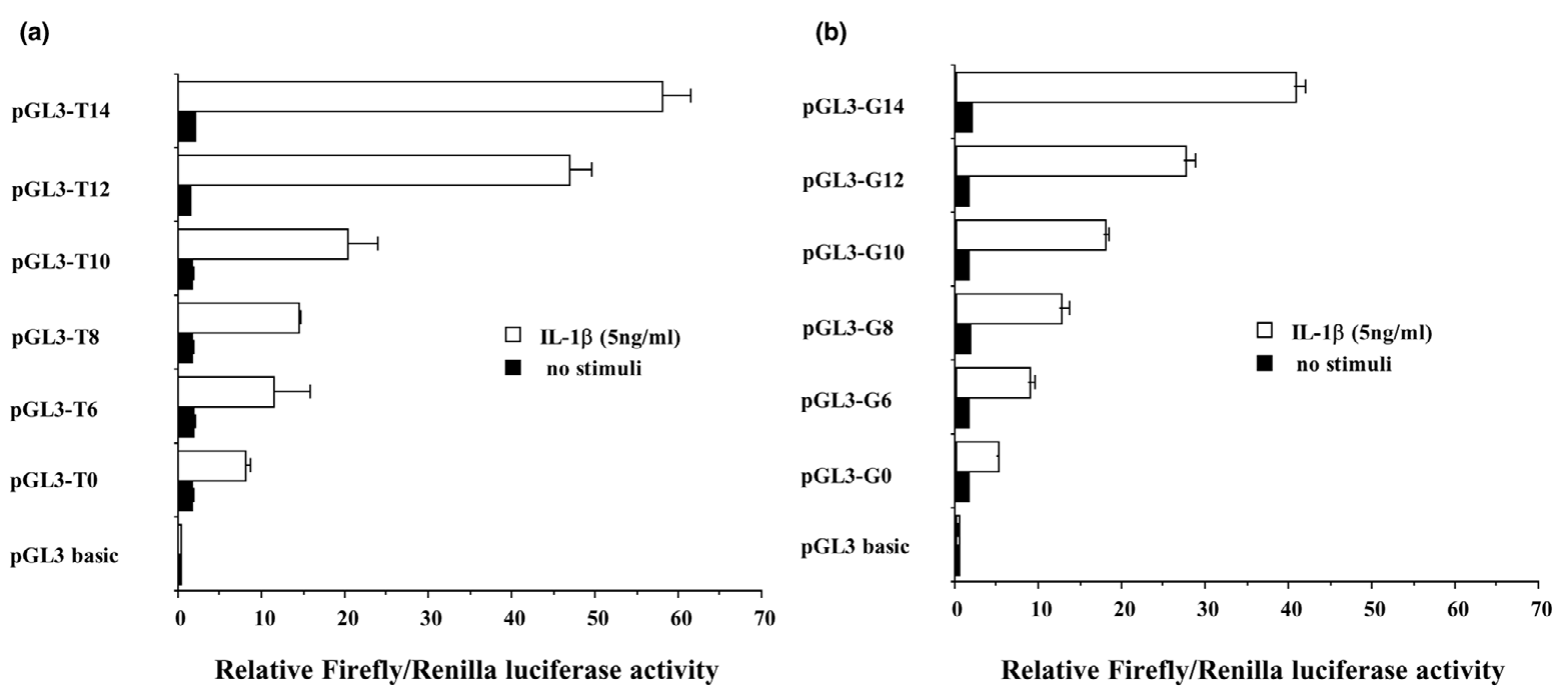
Luciferase reporter assay of the *NOS2 *promoter with gene polymorphisms. The **(a) **pGL3-T vectors and **(b) **pGL3-G vectors were cloned by the *NOS2 *promoter region, including alleles T and G of the single nucleotide polymorphism at -1026 bp. In the series of pGL3-T or pGL3-G vectors, the 6, 8, 10, 12 and 14 repeats of the CCTTT region were cloned upstream of the inserted *NOS2 *promoter in pGL3-T or pGL3-G. bp, base pairs; IL, interleukin.

To determine whether the polymorphisms of -277 SNP and -1,026 SNP and variable numbers of tandem repeat were associated with transcription of the *NOS2 *gene, we evaluated promoter activities using the series of *NOS2 *promoter-luciferase constructs (as described under Materials and method, above). As shown in Figure 5, *NOS2 *was almost transcriptionally silent in fibroblasts without stimuli. In contrast, transcription was induced in fibroblasts transfected with vectors, including promoter regions of the *NOS2 *gene, under stimulation by interleukin-1β. The relative luciferase activities gradually increased with increasing number of CCTTT repeats in both alleles G and T at -1,026. In the case of the same number of CCTTT repeats, the relative luciferase activity was higher in vectors that included the promoter region with allele T at -1,026 than in vectors that included the promoter region with allele G. These findings indicate that transcriptional activity of the *NOS *gene that includes G at -1,026 and a small number of tandem repeats was low.

## Discussion

In the present study were found that concentrations of NO metabolites were not increased in patients with both SSc and PAH, although plasma ET-1 levels were markedly elevated. Our previous report [21] indicates that serum levels of NO metabolites were significantly higher in patients with SSc than in healthy control individuals, especially in patients with a diffuse cutaneous type, active fibrosing alveolitis, or a short duration since onset. However, the population considered in that study did not include patients with PAH, which could explain why the present findings are inconsistent with those of the previous report. Although a number of reports have been published concerning concentrations of ET-1 or NO in the circulation of patients with SSc [8,13-21], this report is the first to describe an imbalance in the NO/ET-1 ratio in patients with PAH.

Over the past decade abnormalities in NO synthesis have been proposed as being important in the pathogenesis and development of pulmonary hypertension, especially primary pulmonary hypertension (PPH). Initially, immunohistochemical studies showed that pulmonary hypertension was associated with diminished expression of NOS-3 [37]. However, other studies found increase in expression of NOS-3 in patients with pulmonary hypertension and in animal models of pulmonary hypertension [38,39]. Despite these contradictory findings, it has been reported that NO levels in blood and the lungs were precisely decreased in patients with PPH and collagen disease related PAH [8-12]. Furthermore, it was determined that NOS-dependent endogenous NO synthesis was decreased in patients with PPH, which suggests that NOS activity may be diminished in patients with PPH [40]. Lung inflammation leading to increased levels of cytokines and oxidants may contribute to the development of both PPH and SSc-related PAH [41]. In the presence of increased levels of inflammatory mediators, NOS activity may be dependent on production of NOS-2, which is distinct from NOS-3 (the endothelial form of NOS) because NOS-2 is inducible by inflammatory mediators, and induced levels are much greater than levels of constitutive NOS-3 production. Peripheral mononuclear cells and lesional fibroblasts are capable of aberrant production of inflammatory cytokines in patients with SSc [42-44]. These cytokines may be involved not only in ET-1 synthesis by endothelial cells and fibroblasts but also in induction of NOS-2. Also, excessive production of ET-1 can mediate NOS-2 production through ET receptor B [45]. Although evidence based on those biological properties may promote speculation that levels of ET-1 correlate with levels of NO in the circulation, NO metabolite levels were within normal range in patients with both SSc and PAH patients whose serum contained much ET-1. We hypothesize that this discrepancy may be explained by reduced NOS-2 production resulting from polymorphisms in the *NOS2 *gene.

As a result of sequencing the promoter region of the *NOS2 *gene from -100 to -1,335, we were able to confirm the presence of two SNPs, consistent with previous reports [46]. In the present study, allele A at -277 SNP, allele G at -1,026 SNP and shorter forms of the CCTTT repeat were associated with susceptibility to PAH combined with SSc. The number of CCTTT repeats was previously reported to influence transcription of the *NOS2 *gene [47]. However, studies of variable numbers of tandem repeat both *in vitro *and *in vivo *have yielded conflicting results [48]. To confirm whether those polymorphisms affect transcription of the *NOS2 *gene in fibroblasts, we constructed a series of luciferase reporter vectors cloned by various numbers of CCTTT combined with the promoter region of the *NOS2 *gene from +58 to -1,557, which included two kinds of haplotype.

Transcriptional activity was lowest in the *NOS2 *gene containing the six repeats of CCTTT and haplotype GA, which suggests that transcription of the *NOS2 *gene might be little induced by interleukin-1β in patients with SSc-related PAH.

Irrespective of whether patients with SSc had PAH, CCTTT repeat length was well correlated with NO/ET-1 ratio. With regard to the relationship between CCTTT repeat length and serum NO levels, we found no significant difference among SSc patients without PAH, although there were significant differences among all SSc patients and among patients with both SSc and PAH (data not shown). In the setting of aberrant production of ET-1 or cytokines, NO synthesis via NOS-2 induction may be dependent on *NOS2 *gene polymorphisms. In healthy control individuals, who had no vascular damage, inflammation, or autoimmune disorders, there was no association between CCTTT repeat length and either serum NO levels or NO/ET-1 ratios (data not shown). Because NOS-2 induction is well controlled by ET-1 and cytokines, distinct from NOS-3, which is constitutively produced, it has been suggested that the CCTTT repeat length is more significantly correlated with NO/ET-1 ratios than with serum NO levels. Our observations support the concept that the *NOS2 *gene polymorphism is a crucial factor in NO synthesis under conditions of vascular damage and chronic inflammation, as well as PAH.

It is not possible to determine whether SSc patients without PAH will suffer this complication in the future, and this is a limitation of the present study. The patients enrolled in the study are from a prospective cohort at our institution, and they have been observed for clinical complications, including PAH, in the follow-up clinic. None of the 58 patients with SSc but not PAH has yet been diagnosed with PAH (mean duration of observation: 45 months).

## Conclusion

NO is a key factor in generating PAH complicated by SSc, and the decrease in NO synthesis might be attributable to reduced NOS-2 production, which is dependent on *NOS2 *gene polymorphisms. Therapeutic options for PAH occurring as a complication of SSc are limited; however, it is not usually the first complication, and it develops several years after SSc is diagnosed. We believe that the development of means to predict the occurrence of PAH related to SSc, and hence prevent this complication, would be a great step forward. Although prospective, longitudinal studies are needed, we propose that patients with SSc who exhibit an imbalance between NO and ET-1 production and who have a short length of CCTTT repeat of the *NOS2 *gene can be treated with a phosphodiesterase type 5 inhibitor before the occurrence of PAH.

## Abbreviations

bp = base pairs; DMEM = Dulbecco's modified Eagle's medium; ET = endothelin; FBS = foetal bovine serum; NO = nitric oxide; NOS = nitric oxide synthase; PAH = pulmonary arterial hypertension; PCR = polymerase chain reaction; PPH = primary pulmonary hypertension; SNP = single nucleotide polymorphism; SSc = systemic sclerosis.

## Competing interests

The authors declare that they have no competing interests.

## Authors' contributions

YK designed the study, recruited the patients and drafted the manuscript. AT was responsible for the recruitment and classification of the patients, and determined genotypes of *NOS2*. MH participated in coordination of the study. MK determined the phenotype of polymorphisms. TS and YK participated in coordination of the study. JO, HK and MO were responsible for the recruitment and classification of patients and healthy volunteers. NK participated in the design and coordination of the study. All authors read and approved the final manuscript.

## Figures and Tables

**Table 1:** Clinical characteristics of patients

Characteristic	SSc patients		Healthy controls
		
	With PAH	Without PAH	
Number at entry (*n *(male:female)	20 (1:19)	58 (5:53)	95 (8:87)
Age (years; mean (range)	48.6 (17–80)	47.7 (19–77)	40.9 (22–78)
Diffuse SSc:limited SSc (*n*)	6:14	33:25	
Disease duration (months; mean (range)	42.0 (6–130)	41.9 (5–120)	
Pulmonary fibrosis (*n *(%)	6 (30)	31 (53)	
Frequency of ANA (*n *(%)			
Anti-U1-snRNP antibody	11 (55)	17 (29)	0
Anti-topoisomerase I antibody	1 (5)	13 (22)	0
Anti-centromere antibody	5 (25)	12 (21)	0

ANA, antinuclear antibody; PAH, pulmonary arterial hypertension; SSc, systemic sclerosis.

**Table 2:** Distribution of single nucleotide polymorphisms in *NOS2 *gene promoter region

Subjects	-1,026 bp			-277 bp			Haplotype			
	
	G/G	G/T	T/T	A/A	A/G	G/G	GA	TA	GG	TG
SSc with PAH (*n *= 20)	20 (100)	0	0	20 (100)	0	0	40	0	0	0
SSc without PAH (*n *= 58)	43 (74)	11 (19)	4 (7)	43 (74)	10 (17)	5 (9)	96	0	1	19
Healthy controls (*n *= 95)	69 (73)	23 (24)	3 (3)	73 (77)	19 (20)	3 (3)	161	0	4	25

Values indicate number (%) of genotype or number of haplotype, which consists of two SNPs at -1,026 and -277. There were significant differences between patients with SSc who did and those who did not have PAH in the distribution of two SNPs at -277 and -1,026 (both *P *= 0.04, by Fisher's exact test). Comparing the distribution of two SNPs between patients with SSc complicated by PAH and healthy control individuals, there was a significant difference at -1,026 (*P *= 0.02), but there was no difference at -277 (*P *= 0.053). With respect to haplotype, the frequency of GA was significantly higher in SSc with PAH than in SSc without PAH and in healthy control individuals (*P *= 0.001 and *P *= 0.02, respectively). PAH, pulmonary arterial hypertension; SNP, single nucleotide polymorphism; SSc, systemic sclerosis.

**Table 3:** Allele frequencies for the CCTTT-repeat polymorphism

Allele	SSc patients		Healthy controls
		
	With PAH	Without PAH	
6	2	0	0
7	2	0	0
8	8	0	4
9	6	4	8
10	10	11	19
11	5	16	25
12	6	29	42
13	0	22	32
14	1	14	29
15	0	7	13
16	0	4	13
17	0	4	4
18	0	2	1
19	0	2	0
20	0	0	0
21	0	1	0

*P *< 0.0001 for patients with both SSc and PAH compared with healthy control subjects by the Mann-Whitney *U *test. *P *< 0.0001 for patients with both SSc and PAH compared with patients with SSc but not PAH by the Mann-Whitney *U *test. PAH, pulmonary arterial hypertension; SSc, systemic sclerosis.
